# Perioperative risk factors related to complications of lumbar spine fusion surgery in elderly patients

**DOI:** 10.1186/s12891-023-06689-z

**Published:** 2023-07-14

**Authors:** Chenjun Liu, Chen Guo, Fanqi Meng, Zhenqi Zhu, Weiwei Xia, Haiying Liu

**Affiliations:** Department of Spinal Surgery, PeKing University People’s Hospital, 11th. Xizhimen South Ave., Beijing, 100044 China

**Keywords:** Elderly, Lumbar spine, Surgery, Risk factors, Complication

## Abstract

**Purpose:**

To analyze the perioperative risk factors related to lumbar spine fusion surgery in elderly patients.

**Methods:**

202 elderly patients (age range 77–92 years old) who have underwent lumbar spinal fusion surgeries between January 2019 and June 2021 were retrospectively investigated. Information of age, sex, comorbidity, fixation segments, operation time, surgical blood loss and perioperative complications during hospitalization were collected. Risk factors for complications were analyzed. Student’s *t*-test, *chi-square* test, Mann-Whitney *U*‑test and multivariate generalized linear models were used.

**Results:**

In this study, 31 patients presented complications (15.3%) in these elderly patients with an average age of 79.1 years, including 1 patient with intraoperative complication and 30 patients with postoperative complications; and 2 out of 31 patients (1%) died. The elderly patients were divided into group A (24 patients) with major postoperative complications and group B (178 patients) without major postoperative complications. Major postoperative complications were significantly associated with age (univariate analysis, *t* = 3.92, *P* < 0.001; multivariate analysis, *OR* = 1.323, *95%CI* 1.126–1.554, *P* = 0.001), but not significantly associated with other factors tested (sex, comorbidity, fixation segments, operation time, surgical blood loss). Then 173 patients (range 77–81 years) were selected and the rate of major postoperative complications of each age from 78 to 81 years was compared with that of 77 years patients, respectively. We found that the ratios of complications at 80 years (*OR* = 10.000, *P* = 0.019) and 81 years (*OR* = 10.000, *P* = 0.009) were higher than the ratio at 77 years.

**Conclusions:**

Although with great progress of medical technology, increasing age was still the independent risk factor for major postoperative complications in elderly patients undergoing lumbar spinal fusion surgery. As for the incidence of major postoperative complications, 80 and 81 years old patients was 10 folds higher than that of 77 years old patients, reminding us to pay more attention to 80 years old and even older patients.

Currently, the global population is experiencing a shift in its age structure due to decreasing fertility rates and increasing longevity. The World Health Organization has given new standards for old age: people between 60 and 74 are called young elderly, and people older than 74 are called elderly. As a result of the population aging, there is a remarkable change in the cause of disease and mortality. This is referred to the “epidemiological transition” and reflects the evolution from infections, parasitic and nutritional deficiency diseases to chronic and degenerative diseases [1]. Although spinal disorders are not typically regarded as life threatening, they can lead to chronic pain and significant limitation of activity, which will extensively affect working ability and the quality of life [2, 3].

An accurate assessment of the potential morbidities associated with spinal procedures is necessary to guide treatment. The symptomatic lumbar spinal disease should be treated individually irrespective of patient age. Conservative treatment such as medication, physical therapy, and steroid injection may relieve the symptoms in some cases [4]. However, surgical treatment, and even fusion surgery, is typically needed for medically refractory cases with moderate to severe lumbar spinal disease. Posterior lumbar interbody fusion (PLIF) is a common technique for treating degenerative lumbar diseases. PLIF involves the effects to decompress the neural elements and stabilize the affected segment [5]. PLIF has generally been indicated for patients younger than 70 years old, however, it is being performed increasingly for patients older than 70 years old which may due to the improved technique of surgeons, medical treatments and devices [6–8].

Spinal surgery in the elderly has historically been thought to be associated with an increased risk for perioperative complications [7, 8]. But several studies have shown that elderly patients have similar complication rates and clinical outcomes after lumbar decompression and/or fusion procedures compared with younger patients [9–12]. It is an important issue for spinal surgeons to ensure the safety, as well as the effectiveness of geriatric patients’ surgeries. It is generally accepted that older age, longer operation time, more surgical blood loss and larger surgeries might lead to higher risks for complications. However, what’s the fact indeed? Especially for patients of 80 years old or even older, does the increased age affect the risks for lumbar spine fusion surgery? Therefore, the present study aimed to analyze the perioperative risk factors related to lumbar spine fusion surgery in elderly patients.

## Materials and methods

### Study design and inclusion criteria

In this study, 202 patients (age range 77–92 years old) who were underwent posterior lumbar intervertebral fusion surgery from January 2019 to June 2021 were selected. The inclusion criteria were (1) age of patients > = 77 years old (2) patients were underwent posterior lumbar intervertebral fusion surgery in our department from January 2019 to June 2021. The exclusion criteria were (1) patients with traumas or tumors (2) patients with previous history of surgery. Data of age, sex, comorbidity, fixation segments, operation time, surgical blood loss and perioperative complications were collected in this study.

The study was conducted under the Declaration of Helsinki and was approved by the Ethics Committee of Peking University People’s Hospital (No. 2019PHB186‑01). Informed written consent was obtained from all patients before their enrollment in the study.

### Research data

A complication was defined as any event requiring specific management during the perioperative period, including the intraoperative and postoperative periods. Postoperative complications were further classified as major (adversely affecting the recovery of the patient) and minor (recorded in the medical chart but did not alter the recovery of the patients). In our study, major postoperative complications included coronary artery disease/congestive heart failure/cardiac insufficiency, pneumonia, wound infection (debridement), severe arrhythmia, neurological dysfunction (transient foot drop), athma, Guillain-Barre syndrome, acute cerebral infarction and deep venous thrombosis; minor postoperative complications included delirium, atrial fibrillation and incomplete intestinal obstruction.

These elderly patients were divided into two groups: Group A with major postoperative complications and Group B without major postoperative complications. Age, sex, comorbidity, fixation segments, operation time and surgical blood loss were compared between these two groups. Then to further study the risk of aging to complications, patients from 77 to 81 years were selected and rate of major postoperative complications of each age was compared with that of 77 years patients, respectively.

Comorbidities were subdivided as following: cardiovascular disease (hypertension, arrhythmia, coronary artery disease, and congestive heart failure), pulmonary disease (lung abscess, tuberculosis, chronic obstructive pulmonary disease, asthma, pneumonia and sleep apnea syndrome), endocrine disease (diabetes mellitus, hypothyroidism and hyperthyroidism), nervous system disease (cerebral infarction, cerebral hemorrhage and Parkinson’s disease), urinary disease, hepatic diseases, and so on [13].

### Statistical analysis

All statistical analyses were performed with SPSS statistical software version 20.0 (SPSS Inc., Chicago, IL, USA). Descriptive statistics were displayed as mean ± standard deviation (SD). Several univariate statistical analyses were performed to estimate the association among various factors as following: to determine the complication difference between two groups, Student’s *t*-test for age; *chi-square* test for comorbidity and sex, Mann-Whitney *U*‑test for fixation segments, operation time, surgical blood loss. Univariate and multivariate logistic regression analysis were used to identify factors associated with complications in elderly patients. Multivariate generalized linear models were applied to assess the rate of major postoperative complications in 77 years patients and older (78–81 years) patients, respectively. For each variable, *OR*s (odds ratios) and *95% CI*s were calculated. A *P* < 0.05 was considered statistically significant.

## Results

### General data

**Fig. 1:**
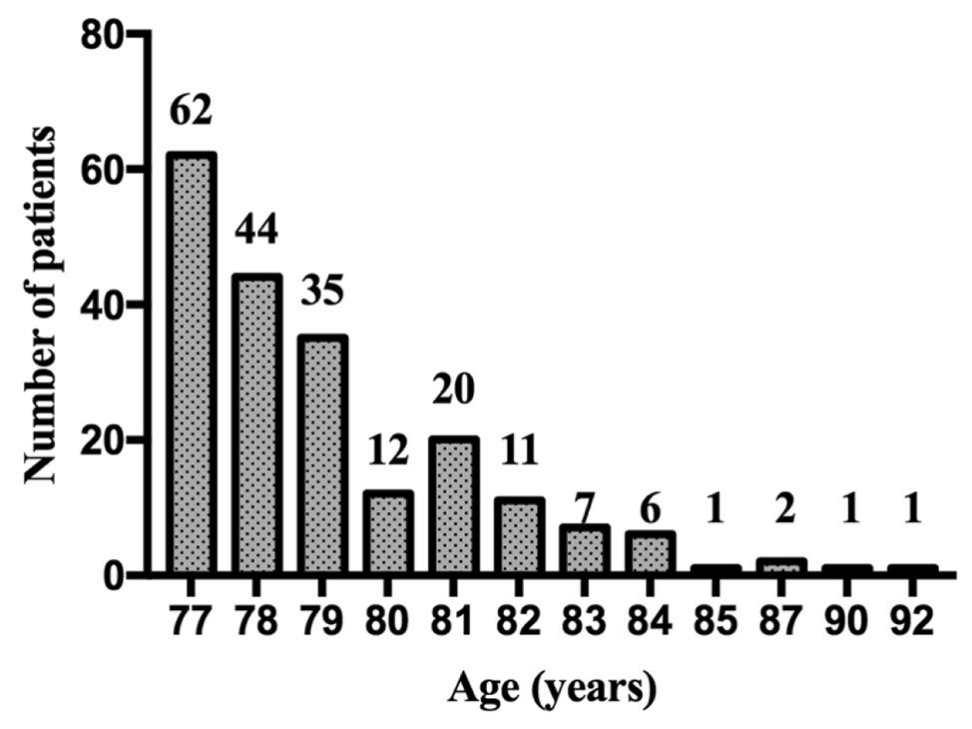
Bar graph showing the population stratified by age (total number of patients was 202)

During the study period from 2019 to 2021, 202 patients, with the average age of 79.1 years (range 77–92 years), underwent instrumented fusion surgery (Fig. 1). In this study, 31 patients (15.3%) presented complications in these elderly patients, including 1 patient with intraoperative complications and 30 patients with postoperative complications (24 patients with major complications and 6 patients with minor complications). Moreover, 2 patients of them (1%) died (Table 1). Patient 1, male, 81 years old, died of postoperative acute myocardial infarction and arrhythmia. Patient 2, female, 78 years old, died of postoperative acute myocardial infarction, heart failure, respiratory failure and pulmonary infection.

**Table 1:** Complications of lumbar spine fusion surgery of elderly patients in our research

Complications		Patients
Major postoperative complications	Coronary artery disease/Congestive heart failure/Cardiac insufficiency	10
	Pneumonia	7
	Wound infection (debridement)	3
	Severe arrhythmia	2
	Neurological dysfunction (transient foot drop)	1
	Athma	1
	Guillain-Barre syndrome	1
	Acute cerebral infarction	1
	Deep venous thrombosis	1
Minor postoperative complications	Delirium	4
	Atrial fibrillation	3
	Incomplete intestinal obstruction	1
Intraoperative complications	Cerebrospinal fluid leakage	1
	Multiple	5

In these patients, 99 patients (49%) were male and 103 (51%) were female. Mean operation time was 153 minutes and mean surgical blood loss was 365 ml. Most of major postoperative complications (87.5%) occurred in the first five days after operations.

### Risk factors for postoperative complications

These elderly patients were divided into two groups: group A (24 patients) with major postoperative complications and group B (178 patients) without major postoperative complications. There were significant differences of age between two groups (univariate analysis, *t* = 3.92, *P* < 0.001; multivariate analysis, *OR* = 1.323, *95%CI* 1.126–1.554, *P* = 0.001). However, major postoperative complications were not significantly associated with other factors tested (sex, comorbidity, fixation segments, operation time, surgical blood loss) (Table 2).

**Table 2:** Results of univariate and multivariate analysis about perioperative risk factors related to complications of lumbar spine fusion surgery

Parameters	Group A(24 patients)	Group B(178 patients)	Univariate	Multivariate
Age(years)	80.88 ± 3.18	78.87 ± 2.23	*P* < 0.001* *t* = 3.92	*P* = 0.001† *OR* = 1.323 *95%CI* (1.126–1.554)
Comorbidity	19/24 (79.2%)	132/178 (74.2%)	*P* = 0.596 *χ*^*2*^ = 0.281	*P* = 0.441 *OR* = 1.557 *95%CI* (0.505–4.803)
Sex(male)	13/24 (54.2%)	86/178 (48.3%)	*P* = 0.290 *X*^*2*^ = 0.281	*P* = 0.679 *OR* = 1.227 *95%CI* (0.466–3.234)
Fixation segments				
1	4	33	*P* = 0.848 *Z*=-0.192	*P* = 0.171 *OR* = 1.466 *95%CI* (0.848–2.535)
2	13	88		
3	4	33		
4	1	17		
5	2	5		
6	0	1		
7	0	1		
Operation time(min)	147.9 ± 33.7	154.0 ± 43.1	*P* = 0.785 *Z*=-0.272	*P* = 0.474 *OR* = 0.994 *95%CI* (0.978–1.010)
Surgical blood loss(mL)	349.2 ± 186.0	367.2 ± 232.2	*P* = 0.971 *Z*=-0.036	*P* = 0.809 *OR* = 1.000 *95%CI* (0.997–1.002)

* Student’s *t*-test † multivariate logistic regression analysis

### Preoperative comorbidities

30.69% of the patients had more than one comorbidity (Table 3). Among the observed comorbidities the most common problem was hypertension and other cardiovascular diseases, which were present preoperatively in 53.96% and 22.77% of the patients. Cardiovascular diseases were subdivided into arrhythmia (8.91%), coronary artery disease (13.86%), and congestive heart failure (0%). Other comorbidities were pulmonary disease (7.1%), urinary disease (3.96%), endocrine disease (20.3%), hepatic disease (2.0%), nervous system (peripheral nerve, 0.6%) and nervous system (brain, 6.93%).

**Table 3:** Preoperative comorbidities of these elderly patients in our research

Comorbidity	Patients
Hypertension	109(53.96%)
Cardiovascular disease	46(22.77%)
Arrhythmia	18(8.91%)
Coronary artery disease	28 (13.86%)
Congestive heart failure	0
Urinary disease	8(3.96%)
Nephrotic cancer	1
Ureter stone	1
Acute renal failure	0
Chronic renal failure	5
Renal cyst	1
Pulmonary disease	11(7.1%)
Lung abscess	0
Tuberculosis	0
Chronic obstructive pulmonary disease	2
Asthma	2
Pneumonia	6
Sleep apnea syndrome	1
Endocrine disease	41(20.3%)
Diabetes Mellitus	35(17.33%)
Hyperthyroidism	1
Hypothyroidism	5
Hepatic disease	3(2.0%)
Common bile duct stone	0
Hepatitis	3
Liver cirrhosis	0
Nervous system(peripheral nerve)	1(0.6%)
Nervous system(brain)	14(6.93%)
Cerebral infarction	13
Cerebral hemorrhage	0
Parkinson’s disease	1
Multiple	62(30.69%)

### Risk of aging to complications

**Fig. 2:**
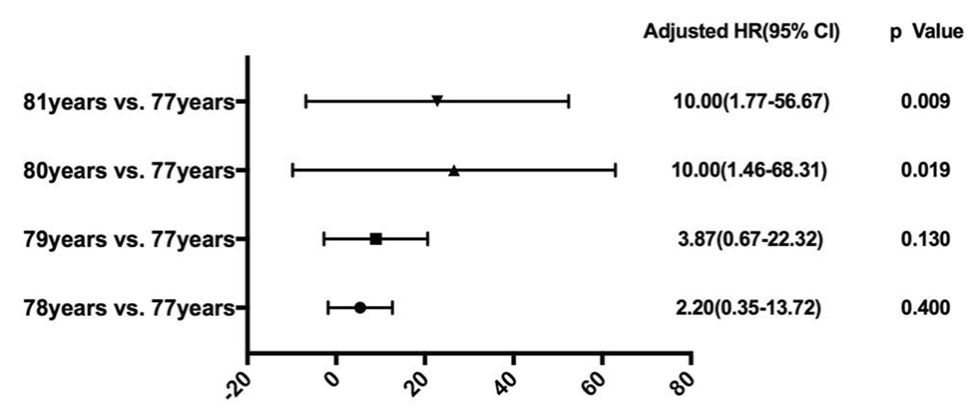
Ratio of major postoperative complications compared in 77–81 years old patients

To further study the risk of aging to complications, 173 patients from 77 to 81 years were selected and rate of major postoperative complications of each age was compared with that of 77 years patients, respectively. The number of patients with major postoperative complications in total number of patients at each age were 2/62 for 77 years, 3/44 for 78 years, 4/35 for 79 years, 3/12 for 80 years and 5/20 for 81 years. The results were listed in (Fig. 2). Compared with ratio of major postoperative complications in 77 years old patients, 80 and 81 years old patients were 10.0 times, while 78 and 79 years old patients showed no statistical differences.

## Discussion

Population aging is nowadays a critical problem that will affect people all over the world. It is predicted that by 2050, the population of the elderly people will be equal to the population of the younger for the first time. There will be two billion people aged 60 or over and another two billion under age 15 [14]. The number of elderly persons in China also continues to grow, with an expected 329 million older than 65 years and 120 million older than 80 years by the year 2050. Along with an aging population, there is an associated increase in age-related diseases. Spinal disease is one of the most critical problems, which seriously restricts people’s activities and then reduces patients’ quality of life. Nowadays, elderly people have longer life, have more active lifestyles, and have a great desire to be pain free throughout the last decades of life. Advances in anesthesiology, spinal instrumentation, and postoperative care have made spinal procedures safer with decreased morbidity and mortality and improved clinical outcomes [15]. Additionally, these advances make it possible to perform more extensive and complex procedures in at-risk populations, such as in elderly patients [16]. Identifying predictors of complications or poor outcomes in the geriatric population is important for perioperative risk assessment and for implementing appropriate preventative treatments.

The relationship between age and safety of spinal fusion surgery has historically been controversial. Investigators have found that patients aged 80 and older experience a striking increase in morbidity and mortality when undergoing spine surgery, with mortality approaching 10% [17, 18]. A study about spinal fusion in 20 patients aged 80 and older showed a 35% major complication rate, which is significantly higher than reported rates in younger patients undergoing similar procedures [19]. However, Okuda et al. found that patients older than 70 years who have undergone posterior lumbar interbody fusion for spondylolisthesis had a 16% of complication rate, which was not significantly different from that in younger patients, but these authors admitted that minor complications were excluded from their analysis [10]. Kilinçer et al. also found that age did not affect the complication rates of posterior lumbar interbody fusion, but they did not report a complication rate separately for older patients [9].

In these elderly patients with an average age of 79.1 years, 31 patients presented complications (15.3%); moreover, 24 patients presented major postoperative complications (11.9%), which is lower than that in several previous retrospective studies [7, 20]. In our study, we found that increasing age was an independent risk factor for major postoperative complications in patients undergoing lumbar spinal fusion surgery whereas other factors were not significant, which was meaningful for surgical therapy of geriatric patients in future. Some preoperative comorbidity-free geriatric patients underwent small spinal surgeries unfortunately developed severe complications although their operation time were short and the surgical blood loss were of a small amount. Therefore, we should pay more attention to all elderly patients and make detailed and thorough surgical plans.

In our research, we collected and analyzed clinical data of geriatric patients with the average age of 80 years, which was more typical than other related studies. Furthermore, we found that 80 years and 81 years patients were 10 folds higher than that of 77 years patients, which quantified the probability of complications significantly increased with age in elderly patients. Oldridge et al. [21] showed an overall mortality rate of 1.3% for 34,418 Medicare beneficiaries undergoing lumbar spine surgery, which was twice than the previously published rates. Besides, the mortality rate of spinal fusions exceeded 10% for patients older than 80 years, and 80–85 years old was identified as a threshold for a dramatic increase in morbidity and mortality. From the perspective of complications, our research remind us in the background of aging society in China, spinal surgeons should pay more attention to 80 years old and even older patients, and comprehensive preoperative preparations and postoperative treatments should be required.

Based on the concept of enhanced recovery after surgery (ERAS), we have adopted several strategies empirically in order to minimize perioperative complications. For the clinical practice of operations on degenerative spinal diseases, some key points were concluded as bellows:

1. The reserved body function is low in old patients, and relative stable conditions can be translated into unstable conditions after certain stimulation. This can easily result in various complications or even death. Therefore, more comprehensive preoperative examinations and evaluations are required. Furthermore, patients over 80 years or accompanied with several comorbidities are usually sent back to intensive care unit, for more deeper care and timely management of possible complications at early stage [22].
2. Doctors should strictly perform medical treatments according to indications. Meanwhile, uncontrolled heart failure, severe arrhythmia and hypertension, serious hepatic and renal insufficiency should be listed as contraindications [23].
3. According to specific conditions of each patient, doctors should not only ensure the safety and effectiveness, but also simplify the surgery procedures and shorten the operation time to reduce the impact of surgical trauma and unwilling physical response. Otherwise, complications and mortality would markedly increase beyond patients’ bearing capacity [24].
4. After the stage of anesthesia, stimulation like pain, drainage tube and body position may induce major change of cardiovascular system, including unstable blood pressure, heart rate and rhythm. For these conditions, appropriate composure and effective analgesia were necessary [25].
5. Active movement of lower limbs and early ambulation should be encouraged to prevent deep venous thrombosis or pulmonary embolism. Frequent roll over and patting on the back, deep breath and cough increase ventilation to prevent accumulation of secretion. Early ambulation could obviously improve cardio-pulmonary function, promote gastro-intestinal motility [26, 27].

Although the findings of this research refer to some important aspects of making surgical strategy, there are several limitations. First, this study is a retrospective analysis. Although we have seriously checked medical records, the retrospective studies may inevitably underestimate the actual complication incidence through the introduction of investigator recall bias. Second, this is a single center study for old Chinese patients, which could not stand for old patients in other regions of China and other countries. Third, the highlight of this study was the patients of older age, but in the following research, we should expand the age range and the follow-up period to further clarify the perioperative risk factors of lumbar spine fusion surgery.

Increasing age was an independent risk factor for major postoperative complications in elderly patients undergoing lumbar spinal fusion surgery whereas other factors were not significant. Considering the rate of major postoperative complications, 80 and 81 years old patients was 10 folds higher than that of 77 years old patients. Although major postoperative complications were indeed less than before, spinal surgeons still should make more comprehensive and detailed therapeutic schedule for elderly patients.

## Data Availability

The datasets generated and analyzed during the current study are available from the corresponding author on reasonable request.

## Abbreviations

PLIF: posterior lumbar interbody fusion
ERAS: enhanced recovery after surgery
